# Reinke's crystals in perivascular and peritubular Leydig cells of men with non-obstructive and obstructive azoospermia: a retrospective case-control study

**DOI:** 10.3325/cmj.2019.60.158

**Published:** 2019-04

**Authors:** Mario Kordić, Davor Tomić, Dragutin Soldo, Dinko Hauptman, Davor Ježek

**Affiliations:** 1 Department of Urology, University Hospital Center Mostar, Mostar, Bosnia and Herzegovina; 2 Department of Urology, University Hospital Center Zagreb, Zagreb, Croatia; 3 Department of Histology and Embryology, University of Zagreb School of Medicine, Zagreb, Croatia; 4 Centre of Excellence for Reproductive and Regenerative Medicine, University of Zagreb School of Medicine, Zagreb, Croatia

## Abstract

**Aim:**

To analyze the differences in the population of perivascular and peritubular Leydig cells (LC) and the number of Reinke's crystals (RCs) in the testicles of infertile men with non-obstructive and obstructive azoospermia.

**Methods:**

This retrospective case-control study was conducted on the testicle tissue of infertile men with obstructive (n = 10) and those with non-obstructive azoospermia (n = 100). Stereological analysis was performed on 7-μm paraffin sections. Measurements were carried out by using the Weibel multipurpose test system.

**Results:**

Patients with non-obstructive azoospermia had a higher total/absolute number of LCs in the perivascular space (*P* = 0.034). In these patients, no significant difference was found in the total and absolute number of RCs between the peritubular and perivascular space. Patients with obstructive azoospermia had around three times higher absolute number of RCs in both the peritubular and perivascular spaces (*P* = 0.002; *P* < 0.001) than non-obstructive group.

**Conclusion:**

Our results suggest that in patients with non-obstructive azoospermia LCs migrated or had different densities in the peritubular and perivascular space compared with patients with obstructive azoospermia. Moreover, the lower number of RCs could imply their utilization by LCs in testosterone production.

Male infertility is defined as an inability to conceive a child over a period of one year with the same partner and with regular and unprotected sexual intercourses. The incidence of infertile couples ranges from 15% to 20%, 20%-50% out of which is related to male infertility (1). Given such a high incidence, infertility takes an important place in several medical branches, and treatment often requires an interdisciplinary approach.

The key organ responsible for man's fertility, as well as its endocrine status, is the testicle. There are two distinct histological and functional compartments, the tubular and interstitial. The tubular part, inside Sertoli cells of the seminiferous epithelium, contains Charcot-Bötcher crystals, while Leydig cells (LC) of the interstitial compartment contain Reinke's crystals (RCs) (2,3). Although they were described by Friedrich Berthold Reinke at the end of the 19th century (3), the knowledge about these crystals is poor.

Aside from the testicle, these crystals were also described in the ovarian hilus cells and adrenal glands, ie, in tissues involved in androgen production (4,5). RCs in men are mostly present after puberty and their number increases with age. This finding resulted in further research on the relationship between these crystals and testosterone concentration. However, it has been proven that the number of RCs did not depend significantly on testosterone concentration (5-8). Recent studies have shown a difference in the number of crystals in various testis pathologies, which led to a using the number of RCs in diagnosing Leydig cell tumors, even though they are present in less than 40% of cases (8). Distribution of RCs is not homogeneous; most LCs do not contain RCs, but some are extremely rich in crystals. Within LCs, RCs are positioned individually, in pairs, or in groups, but they are not present in each cell. They can be found mostly in the cytoplasm, but they also appear in the nucleus. By using histochemical methods, Janko et al (9) demonstrated that RCs consisted of globular proteins positioned in parallel, or less commonly, perpendicularly to each other (9). Ultrastructural data have confirmed the crystals are formed by globular proteins that create a crystal lattice (2).

The material exposed to formalin and various solvents that dehydrate specimens would dissolve phospholipids, glycolipids, and neutral lipids. Thus, a positive reaction after coloring RCs with bromphenophthylene indicates that they are partially or entirely composed of simple proteins (9). A positive reaction to Baker's modification of Millon's dye and p-dimethyl-amino-benzaldehyde nitrite proves that crystals also contain tyrosine and tryptophan (9). Lobo et al have shown that the main protein in RCs is nestin (10). In the most recent studies, nestin is suggested as a marker of angiogenesis, due to the fact that it is expressed in newly formed endothelial cells in the prostate, breast, and other organs, as well as in thyroid cancer (11-13).

Non-obstructive azoospermia (NOA) is one of the most common causes of male infertility, whose pathogenic mechanisms are poorly known. It is clear that the pathogenesis of this disorder is mainly affected by hormone mechanisms, reflecting the changes in the function of LCs. The aim of the study was to analyze the differences in the population of perivascular and peritubular LCs and the number of RCs in the testicles of infertile men with NOA compared with those of men with obstructive azoospermia (OA).

## Participants, methods, and materials

This retrospective case-control study was conducted on the bioptic material of testicles from the archive of the Department of Histology and Embryology at the School of Medicine, University of Zagreb. Bioptic samples from infertile patients with confirmed azoospermia were collected from 1998 to 2006, in collaboration with the Clinic of Urology (University Clinical Center Zagreb) and the University of Zagreb School of Medicine. Bioptic specimens were divided into two groups: one consisted of 10 infertile men with OA and the other of 100 infertile men with NOA. The study was approved by the Ethics Committee of the University of Zagreb School of Medicine (February 17, 2006).

Concerning OA, the study included only the samples with morphologically completely preserved testicle tissue and normal structure. Histological analysis in the study (NOA) group showed a disorder of spermatogenesis, varying in the degree and extent. Patients with cryptorchidism were excluded from the study group, due to certain changes in the number of RCs, the interpretation of which might be misleading.

Samples were obtained by the “open” testicular biopsy method (14). Bioptic parenchyma of the testicles was divided into two parts immediately upon excision and fixed consecutively in 5.5% glutaraldehyde (0.05 M phosphate buffer, pH 7.1-7.4, 800 mosmol) and Bouin. After three-hour fixation, the tissue fixed in glutaraldehyde was rinsed in a buffer, after which the samples were post-fixed in 1% osmium tetroxide solution. After a two-hour post-fixation, the tissue was washed in the buffer and dehydrated in ascending series of alcohols. After dehydration, it was immersed into a mixture of Durcopan resin (Sigma, Neustadt an der Weinstraße, Germany). Resin was polymerized in a thermostat at 60°C during a 3-day period. After polymerization, the blocks were cut by an ultramicrotome (Reichert, Vienna, Austria). The tissue fixed in Bouin was washed out, dehydrated through an ascending series of alcohol, and embedded into paraffin.

Serial semi-thin sections, about 0.90 μm in thickness, were placed on the slides and dried using a warm cutting table (Agar Scientific Ltd, Stansted, UK). Each sample was sliced to 6 item slides, which had a minimum of 10 semi-thin sections. Semi-thin sections were stained with 1% toluidine blue. Serial pairs of 7-μm thick paraffin sections were then placed on 6 slides with a minimum of 10 sections, which were then stained with hemalaun-eosin and the modified Masson method.

### Stereological analysis

Stereological analysis was performed on 7 μm-thick paraffin sections and 0.9 μm-thick semi-thin sections. For the purposes of stereological measurement, only every fourth section cut was chosen out of the total of 60 serial sequences obtained from each bioptic sample, by using the principle of a physical dissector (15,16).

Measurements were carried out by using the Weibel multipurpose test method (17) with 42 test points and a binocular microscope with a total magnification of ×1000. Test area was 0.0144 mm^2^. Sixty fields were analyzed for each patient.

The following variables were determined during stereological analysis: number of hits on peritubular LCs in the test field; number of hits on perivascular LCs in the test field; number of peritubular LCs: relative values and absolute values; number of perivascular LCs: relative values and absolute values; number of RCs in the peritubular space: relative values and absolute values; number of RCs in the perivascular space: relative values and absolute values. Relative values are the number of examined structures in the volume unit and absolute values are the total number of structures in the whole organ. The number of hits refers to the number of testing system points (out of a total of 42) that fall into the studied phase (LCs) from which the volume density was calculated according to Thomson and Glagolev (18,19).

### Statistical analysis

In this case-control study, a total of 100 NOA samples were compared to a total of 10 OA samples as controls. Normality of the distribution of numeric variables was tested with the Shapiro-Wilk's test. The data concerning the number of LCs and RCs was logarithmically transformed. Normally distributed numerical data are expressed as mean values and standard deviations. Age is expressed as median and range. The differences of normally distributed numeric variables between the two independent groups were tested by the Welch's *t* test, and Mann-Whitney's U test, where appropriate. All *P* values were two-sided. The level of significance was set to 0.05. The analysis was performed by using Statistica software package, version 13.2 (Dell Inc., Round Rock, TX, USA).

## Results

**Figure 1:**
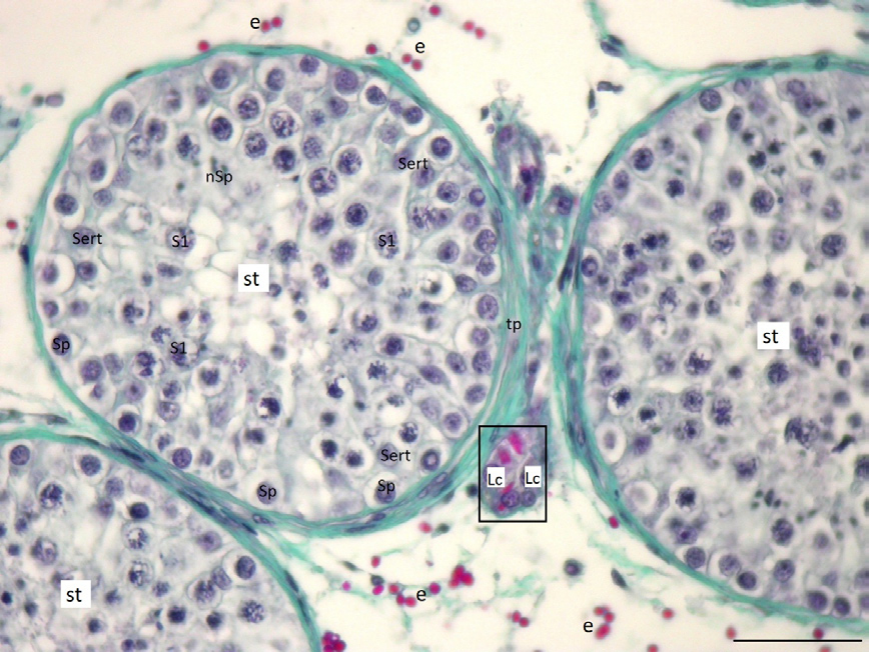
The testis tissue in a patient with obstructive azoospermia. Seminiferous tubules (st), limited by the tunica propria (tp), have a regular diameter and a fully preserved seminiferous epithelium. The epithelium consists of supporting Sertoli cells (Sert) and all types of spermatogenic cells (spermatogonia, Sp; spermatocytes /predominately primary/, S1; newly formatted spermatids that will be soon transformed into spermatozoa, nSp). Attached to seminiferous tubules is a cluster (squared area) of peritubular Leydig cells (Lc) with many Reinke´s crystals in their cytoplasm. These crystals should not be confused with extravasated erythrocytes (e). Masson trichrome staining, scale = 50 μm.

Qualitative histological analysis of semi-thin sections in patients with OA showed the same image in all bioptic samples. The biopsy tissue sample of the testicle consisted of an average of 15-20 seminiferous tubules with the belonging interstitial. Seminiferous tubules did not alter in the diameter (170-200 μm) and were of a normal lamina (tunica) propria thickness. The lamina propria consisted of 5-7 layers of elongated peritubular (myoid) cells. The seminiferous epithelium of the tubules contained supportive Sertoli cells and all forms of spermatogenic cells: spermatogonia, primary and secondary spermatocytes, round and elongated spermatids, and spermatozoa. Sertoli cells had an oval, round, or pear-shaped nucleus with a frequently well-visible nucleolus. OA group had a high Johnsen's “score” (8-10). Perivascular LCs were slightly distant from the seminiferous tubules and closer to smaller or larger blood vessels and had a round or ovoid shape. Peritubular LCs were in the close proximity or attached to the lamina propria of the tubules and had more elongated shape. Bigger or smaller RCs could be observed in some of the perivascular and peritubular LCs (Figure 1).

**Figure 2:**
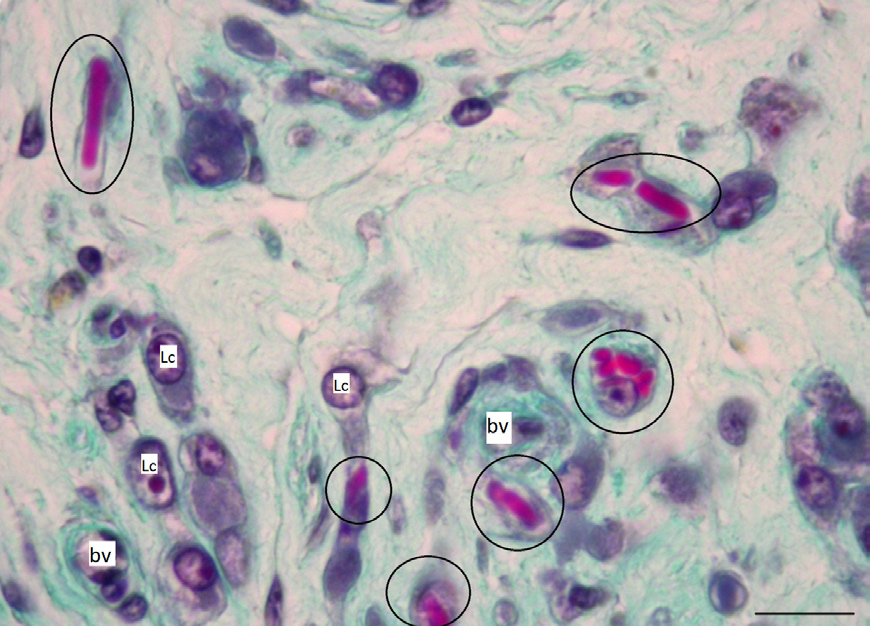
A part of the interstitial tissue in a biopsy of a patient with non-obstructive azoospermia (“Sertoli cells-only syndrome”). In the close proximity of small blood vessels (bv) are clusters of perivascular Leydig cells (Lc). Some of them contain abundant Reinke´s crystals (encircled areas). Masson trichrome staining, scale = 15 μm.

**Figure 3:**
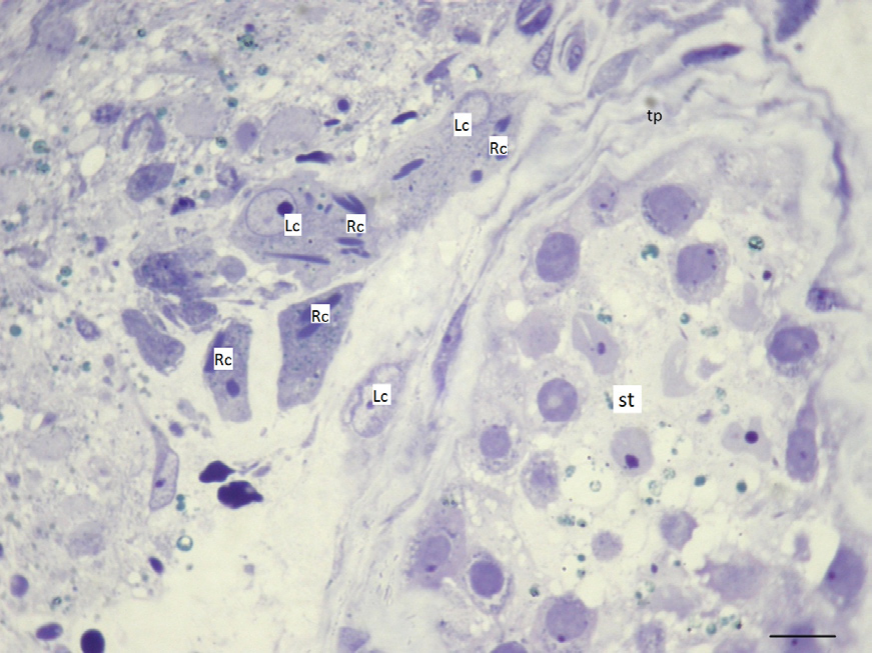
Testicular biopsy from an infertile patient with non-obstructive azoospermia. In the vicinity of a seminiferous tubule (st) are peritubular Leydig cells (Lc) with Reinke´s crystals (Rc) in their cytoplasm. Longitudinally sectioned crystals could reach more than 10 μm in length. The seminiferous tubule is limited by the tunica propria (tp), and the seminiferous epithelium is largely reduced, bearing only spermatogonia and Sertoli cells. Semi-thin section, toluidine blue staining, scale = 10 μm.

**Figure 4:**
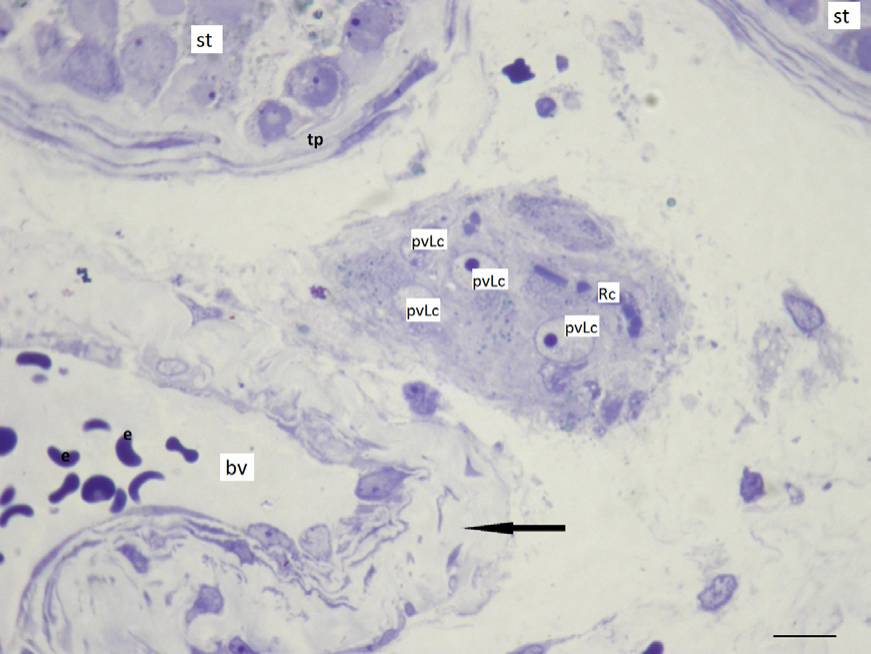
Testicular parenchyma in a patient with non-obstructive azoospermia. Perivascular Leydig cells (pvLc) are located near a blood vessel (bv) and distant to the seminiferous tubules (st) and their border, the tunica propria (tp). In some Leydig cells, different sections of Reinke´s crystals (Rc) can be observed. The wall of the blood vessel (arrow) is limiting the lumen with erythrocytes (e). Toluidine blue staining, scale = 10 μm.

In men with NOA, biopsy specimens consisted of an average of 15-20 seminiferous tubules, and in the cases of greater spermatogenesis impairment (ie, in Sertoli cell syndrome or tubular fibrosis syndrome), around 50 of them. In the biopsy specimens with the Johnsen's “score” of 9-8, seminiferous tubules showed a milder spermatogenesis disorder with the abundant presence of late spermatids and spermatozoa. Biopsy specimens with the “score” of 8-6 had a noticeable lack of spermatozoa and elongated spermatids. Spermatogenesis reached round spermatids stage. LCs retained a typical arrangement around blood vessels and tubules. RCs were sometimes abundant in both individual perivascular and peritubular LCs (Figures 2-4). In some LCs, lower or higher number of vacuoles was visible, while in many of these cells the nucleus and cytoplasm had normal appearance. Within the biopsy samples with the Johnsen's “score” 5-3, the seminiferous epithelium consisted only of supportive Sertoli cells, spermatogonia, and spermatocytes. Along with LCs of normal morphology, smaller or larger groups of hyperplastic and/or hypertrophic cells were observed. RCs were less noticeable, ie, reduced. Due to the impaired spermatogenesis, many tubules were narrowed in the diameter. Spermatogenesis in such seminiferous tubules took place mainly at the level of primary spermatocytes, and apoptotic epithelial cells were often observed inside the lumen of seminiferous tubules. Many of LCs had numerous smaller or larger vacuoles in their cytoplasm and lacked RCs. Biopsy samples with the Johnsen “score” 2 (“Sertoli cells only”) contained narrowed tubules, coated only with supportive cells (Figure 3). The cytoplasm of Sertoli cells was highly vacuolated, and the intercellular spaces were filled with a large number of glycogen granules. The lamina propria was of irregular shape, with a wilted basal membrane. Hyperplastic LCs were present in between the damaged tubules. RCs were scarce but highly recognizable in some LCs (Figure 4). In most severe cases (Johnsen “score” 1), except for seminiferous tubules coated with Sertoli cells only, fibrotic seminiferous tubules were noticed, transformed into rays of connective tissue. The lamina and basal membrane of the seminiferous epithelium were extremely thickened. Due to the extensive remodeling of testicular parenchyma, some LCs were found within fibrous seminiferous tubules. Other LCs were hyperplastic and/or hypertrophic, sometimes with small or larger cytoplasmic vacuoles. Within the loose connective tissue, apart from the changed LCs, an increased number of mast cells and macrophages was observed. Overall, 70% of NOA cases presented histologically as “mixed atrophy” cases, where small groups of seminiferous tubules (or individual tubules) retained preserved spermatogenesis and the production of late spermatids and spermatozoa.

There was no significant difference in the age of patients with OA (median 34, 24-37) and NOA (median 32, 22-43) (Mann-Whitney U test, U = 0.861, *P* = 0.391). No missing data were present.

Men with NOA, compared with men with OA, had significantly lower relative number of LCs in the perivascular space (*P* < 0.001), and somewhat higher number in the peritubular space, but the latter difference was not significant (*P* = 0.089). These values represent the numerical density per mm^3^, which is defined as the relative value (Table 1). In men with NOA, the relative number of LCs was higher in the peritubular than in perivascular space (*t* = -4.003; *P* < 0.001). A similar difference was noticed in the total number of LCs. Men with NOA, compared with men with OA, had a significantly higher total number of LCs in the perivascular space (*P* = 0.043), while in the peritubular space the difference was close to the statistical significance (*P* = 0.095) (Table 2).

**Table 1:** The number of peritubular and perivascular Leydig cells in mm^3^ of testicular bioptic samples of men with obstructive (OA) and non-obstructive azoospermia (NOA) (numerical density, n_v_)

Leydig cells (n_vc_ / mm^3^)	Group	Mean ± standard deviation	Standard error of mean	t (Welch's test)	*P*
Peritubular space	OA	2778.079 ± 1465.386	267.541	1.719	0.089
	NOA	4508.462 ± 3139.015	389.346		
Perivascular space	OA	5928.737 ± 3589.173	445.182	-5.863	<0.001
	NOA	3190.233 ± 996.304	181.899		

**Table 2:** The total number of peritubular and perivascular Leydig cells in histological samples of men with obstructive (OA) and non-obstructive azoospermia (NOA)

Leydig cells (10^3^)	Group	Mean ± SD	Standard error of mean	t (Welch's test)	*P*
Peritubular space	OA	28537.575 ± 11331.285	2068.800	1.685	0.095
	NOA	40152.922 ± 21443.596	2659.751		
Perivascular space	OA	30416.550 ± 7820.845	1427.884	2.146	0.034
	NOA	45614.427 ± 22180.487	2751.151		

The total number of RCs in the peritubular LCs, shown as numerical density (or relative value), did not differ between patients with NOA and patients with OA, but the number of RCs in the perivascular space did (*P* = 0.007). In patients with NOA, there was no significant difference between the total number of RCs in the peritubular and perivascular space (*t* = 1.770; *P* = 0.078) (Table 3). The NOA group had the mean volume of testicle of 11 032.310 mm^3^ (SD 6873.525; SE of mean 852.556), which was significantly lower than that in OA group (mean = 20 020.000; SD 554.231; SE of mean 101.188) (*t* = -4.117; *P* < 0.001). There was no difference in the absolute values of the number of peritubular LCs in the entire testicle between the two groups, but after considering the arithmetic mean of the total number of perivascular LCs, a greater number was found in patients with OA (Table 4).

**Table 3:** The total number of Reinke's crystals in the peritubular and perivascular space in histological testicle samples of men with obstructive (OA) and non-obstructive azoospermia (NOA)

Reinke's crystals (10^3^)	Group	Mean ± standard deviation	Standard error of mean	t (Welch's test)	*P*
Peritubular space	OA	851.996 ± 1381.658	460.553	-0.998	0.321
	NOA	647.964 ± 491.019	102.385		
Perivascular space	OA	1227.656 ± 1470.887	490.296	-2.772	0.007
	NOA	824.949 ± 1124.587	234.493		

**Table 4:** The absolute number of Leydig's cells and Reinke's crystals in the peritubular and perivascular space of testicles of men with obstructive (OA) and non-obstructive azoospermia (NOA)

Absolute number of Leydig's cells (10^3^ × mm^3^)	Group	Mean ± standard deviation	Standard error of mean	t (Welch's test)	*P*
Peritubular space	OA	55.512 ± 28.858	5.269	-0.632	0.529
	NOA	46.786 ± 42.584	5.282		
Perivascular space	OA	610.695 ± 165.482	30.213	-2.090	0.040
	NOA	428.220 ± 270.361	33.534		
Absolute number of Reinke's crystals (10^3^ × mm^3^)					
Peritubular space	OA	17.179 ± 28.090	9.363	-3.210	0.002
	NOA	6.771 ± 5.701	1.189		
Perivascular space	OA	24.748 ± 30.005	10.002	-3.625	<0.001
	NOA	8.898 ± 10.379	2.164		

Patients with OA had around three times higher absolute number of RCs in both peritubular and perivascular space (Table 4). In patients with NOA, the absolute number of LCs was significantly higher in the perivascular than in peritubular space (*t* = 24.898; *P* < 0.001), while the number of RCs did not differ between the peritubular and perivascular space (*t* = 1.796; *P* = 0.074).

## Discussion

Our study clearly shows that there is a significant difference between OA and NOA patients in the absolute number of RCs. Men with OA (with high Johnsen's “score”/preserved parenchyma of the testis, including preserved morphology of Leydig cells) demonstrated higher number of RCs when compared to men with NOA cases (low Johnsen's score, impaired spermatogenesis, and frequently changed morphology of Leydig cells). Based on our results, it seems that the normal morphology of the testis also implies regular (high) number of RCs.

NOA is not the most common, but is certainly one of the most difficult forms of male infertility. Approximately 10%-15% of infertile men are diagnosed with azoospermia, out of whom 80 to 85% have the non-obstructive type. Despite the normal serum testosterone values, one part of Leydig interstitial cells in NOA has impaired morphology (20,21). In our study in semi-thin sections of testicles of men with NOA, hypertrophy and possible hyperplasia of LCs were noticed; this is partially confirmed in the available literature (22). Hypertrophy and potential hyperplasia were more frequently present in lower-grade samples (Johnsen's “score” 1-5).

When conducting this study, as part of the stereological analysis of perivascular and peritubular LCs and counting RCs, a different degree of spermatogenesis was observed (Johnsen “score” 1-10). One can assume that LCs from the perivascular space migrate to the peritubular space to enhance testosterone secretion (especially in patients with impaired spermatogenesis) and thus support the process of spermatogenesis. Therefore, the present study aimed to compare the perivascular and peritubular LCs in OA and NOA groups. Considering the average values of relative LCs number within the same space in both groups, a greater number of LCs was noticed in the perivascular than in the peritubular space in patients with NOA, suggesting that in these patients LCs actually migrated.

In patients with NOA, fewer RCs are expected in peritubular and perivascular LCs because it is assumed that these cells mobilize their testosterone production organelles (4,5) as part of their compensatory mechanisms. A number of studies link RCs with steroidogenesis and testosterone metabolism (23,24), Some authors partially link them with pathological occurrences in the testicles, while some do not (25,26). In the same group of patients, hypertrophy and possible hyperplasia of LCs were noticed. The average number of LCs in the unit volume of testicle in the perivascular and peritubular space was higher in the samples with NOA in comparison with the OA group.

Relative and absolute RCs numbers were lower in peritubular and perivascular LCs in patients with NOA than in patients with OA. In the samples with NOA, the number of RCs in the perivascular space was higher than in the peritubular space of LCs.

Recently, RCs have been analyzed in patients with testicular dysgenesis syndrome (TDS) and Leydig cell´s micronodules (27). Micronodules seem to be a compensatory mechanism caused by the androgenic failure. Their formation is presumably driven by high concentrations of luteinizing hormone (LH). In TDS cases and the normally descended testicle, the micronodules contained a paucity of RCs. This could be attributed to recently renewed immature LCs (27). The authors also showed the increased number of RCs in testes that were either undescended at birth or are persistently undescended, which is line with our previous results (28). It seems that RCs are rather sensitive to formalin or alcohol fixation, dissolving rapidly in formalin and slowly and only partially in alcohol (29). Since RCs have only been described in androgen-producing cells, one can assume that, despite the fact that their exact molecular composition is yet not known, the crystals represent a crystallized form of androgenic hormones, hormone complexes, or enzymes involved in their synthesis. Yet, the RCs in that study were only positive to 3β-hydroxysteroid dehydrogenase (3BHSD) (29). The same group of authors immunohistochemically analyzed the tissue and RCs of Leydig cell tumors, testicular adrenal rest tumors, testicular tumors of adrenogenital syndrome, and cases of androgen insensitivity syndrome, demonstrating that RCs represent crystallized forms of a 3BHSD/calretinin complex (30).

Our study has certain limitations: the results are based on a single center experience; there were practical difficulties in the selection and availability of control samples as well as collection of high quality control and pathological samples. Due to all these reasons, there are relatively few available data related to RCs in azoospermia, originating mostly from single-center studies. In general, these data support the concept that RCs are closely connected to androgen synthesis and that they accumulate within LCs. Based on the results presented in our study, it may be proposed that in NOA patients, RCs are somehow “mobilized” by the stimulatory effect of LH and the need for more androgens by degenerating spermatogenic cells. Therefore, one can assume that RCs possibly dissolve in the LCs cytoplasm and release additional testosterone in order to support already deficient spermatogenesis. This concept of RCs “mobilization” and LCs in men with NOA warrants additional research that would elucidate the role of these still mysterious crystals. Additional research would encompass a detailed biochemical analysis of RCs, probably by mass spectrometry and other modern approaches, such as laser microdissection of the cell/isolation of the crystal. In our experience, microdissection and sufficient concentration of the crystal remains the main obstacle for a more thorough biochemical analysis. The biochemical data obtained would finally clarify if the crystals are a by-product of steroidogenesis and the testosterone production or not.
